# Obesity-mediated regulation of cardiac protein acetylation: parallel analysis of total and acetylated proteins via TMT-tagged mass spectrometry

**DOI:** 10.1042/BSR20180721

**Published:** 2018-09-07

**Authors:** Samantha S. Romanick, Craig Ulrich, Karen Schlauch, Andrew Hostler, Jordanna Payne, Rebekah Woolsey, David Quilici, Yumei Feng, Bradley S. Ferguson

**Affiliations:** 1 Department of Agriculture, Nutrition, and Veterinary Sciences, University of Nevada Reno, Reno, NV 89557, U.S.A.; 2 Center for Cardiovascular Research, University of Nevada Reno, Reno, NV 89557, U.S.A.; 3 Department of Pharmacology, University of Nevada Reno, Reno, NV 89557, U.S.A.; 4 Department of Biochemistry and Molecular Biology, University of Nevada Reno, Reno, NV 89557, U.S.A.; 5 Director, NV INBRE Bioinformatics Core, University of Nevada Reno, Reno, NV 89557, U.S.A.; 6 Department of Biotechnology, University of Nevada Reno, Reno, NV 89557, U.S.A.; 7 Nevada Proteomics Center, University of Nevada Reno, Reno, NV 89557, U.S.A.

**Keywords:** acetylation/deacetylation, myocardial remodeling, muscle metabolism, muscle contraction, proteomics

## Abstract

Lysine residues undergo diverse and reversible post-translational modifications (PTMs). Lysine acetylation has traditionally been studied in the epigenetic regulation of nucleosomal histones that provides an important mechanism for regulating gene expression. Histone acetylation plays a key role in cardiac remodeling and function. However, recent studies have shown that thousands of proteins can be acetylated at multiple acetylation sites, suggesting the acetylome rivals the kinome as a PTM. Based on this, we examined the impact of obesity on protein lysine acetylation in the left ventricle (LV) of male c57BL/6J mice. We reported that obesity significantly increased heart enlargement and fibrosis. Moreover, immunoblot analysis demonstrated that lysine acetylation was markedly altered with obesity and that this phenomenon was cardiac tissue specific. Mass spectral analysis identified 2515 proteins, of which 65 were significantly impacted by obesity. Ingenuity Pathway Analysis® (IPA) further demonstrated that these proteins were involved in metabolic dysfunction and cardiac remodeling. In addition to total protein, 189 proteins were acetylated, 14 of which were significantly impacted by obesity. IPA identified the Cardiovascular Disease Pathway as significantly regulated by obesity. This network included aconitate hydratase 2 (ACO2), and dihydrolipoyl dehydrogenase (DLD), in which acetylation was significantly increased by obesity. These proteins are known to regulate cardiac function yet, the impact for ACO2 and DLD acetylation remains unclear. Combined, these findings suggest a critical role for cardiac acetylation in obesity-mediated remodeling; this has the potential to elucidate novel targets that regulate cardiac pathology.

## Introduction

Obesity is a global health problem that is associated with numerous morbidities, including cardiovascular disease. Alarmingly, obesity has increased 28% in adults and 47% in children over the last 30 years [1]. The cardiovascular system responds to body fat accumulation, through a myriad of adaptations that include cardiac tissue remodeling [2]. Remodeling of the myocardium is characterized by increases in ventricular wall thickness and fibrosis, which culminate in the development of diastolic and systolic dysfunction and ultimately heart failure [2–4].

Molecular mechanisms underlying cardiac remodeling includes re-expression of genes normally active during development; this requires ‘unpacking’ of silenced genes via epigenetic mechanisms [3,4] that entails post-translational modifications (PTMs) of DNA or histone proteins. As such, reports have implicated histone acetylation as a key regulator of cardiac remodeling and function [5,6]. While lysine acetylation has traditionally been studied in the context of nucleosomal histones, recent proteomic reports have demonstrated that approximately 4500 proteins can be acetylated on approximately 15000 acetylation sites (e.g. lysine residues), suggesting a more complex role for the acetylome in biological functions [7]. While non-histone protein acetylation remains poorly understood, recent findings in cardiac biology suggest that non-histone acetylation can impact contractile function, energy metabolism, and cardiac remodeling [8–10]. For instance, acetylation of the sarcomeric protein myosin heavy chain was recently reported to increase contractile function in response to stress, whereas deacetylation by histone deacetylase (HDAC) 3 (HDAC3) decreased contractile performance [8].

Contractile performance of the working mammalian heart requires high demand for energy; more than half of these energy needs are met via fatty acid β oxidation followed by glucose oxidation [11,12]. Mitochondria increasingly rely on fatty acid β oxidation in response to obesity for energy utilization with conservation of glucose metabolism [11,12]. Chronically elevated circulating free fatty acids in obesity contribute to increased fatty acid uptake and β oxidation, providing an abundance of acetyl-CoA molecules for protein lysine acetylation [13,14].

Based on reports above, we would postulate marked changes in cardiac protein acetylation associated with obesity. While the cardiac acetylome is an emerging topic of interest [15], our understanding of how obesity regulates cardiac lysine protein acetylation remains inadequate. In this report, we utilized MS/MS to examine the cardiac acetylome in a rodent model of obesity-induced cardiac remodeling. For this report, we relied on tandem mass-tagged (TMT) examination of the proteome and used proteome discoverer software to determine changes in peptide acetylation. Unlike standard enrichment methods that allow for the detection of low abundant peptide modifications, this method afforded us the opportunity to assess peptide acetylation and normalize our findings to total protein expression in one analysis. Through TMT-tagged MS, we identified approximately 2500 proteins in the myocardium, of which 65 were significantly regulated by obesity. In addition, we reported that 189 proteins were acetylated under conditions of diet-induced obesity (DIO), in which 14 proteins were significantly impacted by obesity. Moreover, Ingenuity Pathway Analysis (IPA) identified the Cardiovascular Disease Pathway as significantly impacted by DIO, as 47 of the 189 acetylated proteins identified encompassed this pathway. Of note, acetylated proteins were mostly found to be involved in energy metabolism and muscle contractility, in which acetylation was primarily increased for mitochondrial proteins and decreased for sarcomeric proteins. These findings highlight the potential impact for the cardiac acetylome in the regulation of cardiac pathophysiology.

## Experimental

### Experimental animals

C57BL/6J mice were rendered obese by diet. Diet-induced obese C57BL/6J mice were given *ad libitum* access to a high-fat diet (HFD) consisting of 60% of total kilocalories from fat (Research Diets Inc. D12492) starting at 16 weeks of age, while lean controls were given *ad libitum* access to a low-fat diet (LFD) (control diet (CD)) consisting of 10% of total kilocalories from fat (Research Diet Inc. D12450B). Protein was similar for both diets (10% of total kilocalories). Caloric balance was provided by differences in carbohydrate amount. Mice were fed both diets for a total of 16 weeks. At the end of the protocol, hearts were collected and left ventricles (LVs) dissected for histology and biochemistry. Animal care and use were in compliance with the Institute of Laboratory Animal Research Guide for the Care and Use of Laboratory Animals and approved by the Institutional Animal Use and Care Committee at the University of Nevada Reno. All experiments were in accordance with the National Institutes of Health (NIH) guidelines.

### Real-time qRT-PCR

RNA was isolated from the LVs of CD and HFD male mice using Qiazol (Qiagen) as per manufacturer’s protocol. RNA was quantitated via Nanodrop ND-1000 spectrophotometer. cDNA was reverse transcribed from 500 ng of RNA via Verso cDNA Synthesis Kit (Thermo Scientific; AB-1453). Quantitative PCR (qPCR) amplification was performed on a Bio-Rad CF96X qPCR instrument (Bio-Rad). Mouse primers for qPCR were: atrial natriuretic peptide (ANP) (forward: 5′-GCC GGT AGA AGA TGA GGT CAT-3′, reverse: 5′-GCT TCC TCA GTC TGC TCA CTC -3′), brain natriuretic peptide (BNP) (forward: 5′-CGC TGG GAG GTC ACT CCT AT -3′, reverse: 5′-GCT CTG GAG ACT GGC TAG GAC TT -3′), Collagen 1 (forward: 5′-TTC TAG TTC CTG GGC CTA TCT-3′, reverse: 5′-GAT GCA GGA CAG ACC AAG AG-3′), and connective tissue growth factor 1 (Ctgf-1) (forward: 5′-ACC TGT GCC TGC CAT TAC -3′, reverse: 5′-GTC CCT TAC TTC CTG GCT TTA C-3′) and gene expression was examined and referenced to 18S (forward: 5′-GCC GCT AGA GGT GAA ATT CTT A-3′, reverse: 5′-CTT TCG CTC TGG TCC GTC TT-3′) gene expression. Amplicon abundance was calculated using the 2^−ΔΔ*C*^_T_ method. GraphPad Prism software was used for graph visuals, and included mean ± S.E.M. Statistical analysis was completed by ANOVA followed by post-hoc testing (Tukey’s test was used for post-hoc analysis unless otherwise noted) using GraphPad Prism software. Statistical significance (defined as *P*<0.05) was reported as applicable.

### Histology

LV tissue was fixed in 4% paraformaldehyde and processed using Leica ASP300S and paraffin embedded using Leica EG1160. PicroSirius Red staining was performed on LV tissue cross-sectioned at 5 μm as previously described [16]. Quantitation of collagen present in LV tissue was performed using ImageJ software and sections were imaged using Leica DMI3000B.

### Immunoblotting

LV tissue was homogenized in ice-cold lysis buffer containing PBS (pH 7.4), 0.5% Triton X-100, 300 mM NaCl, and protease/phosphatase inhibitor cocktail (Thermo Fisher) using the Next Advance Bullet Blender. Lysates were clarified at 16000×***g*** for 5 min prior to protein concentration determination via BCA Protein Assay Kit (Pierce). Proteins were resolved by SDS/PAGE, transferred to nitrocellulose membranes (Bio-Rad), and membranes were blocked with 4% milk. Membranes were probed overnight with indicated primary antibodies for mouse monoclonal acetyl-lysine (Cell Signaling Technology; 9681), rabbit polyclonal acetyl-lysine (Cell Signaling Technology; 9441), acetyl-α-tubulin (Santa Cruz Biotechnology; sc-23950), α-tubulin (Santa Cruz Biotechnology; sc-23948), or total histone H3 (Cell Signaling Technology; 4499). Horseradish peroxidase (HRP)–conjugated secondary antibodies (Southern Biotech) were used at a concentration of 1:2000. SuperSignal West Pico chemiluminescence system (Thermo Scientific) and a ChemiDoc XRS+ imager (Bio-Rad) were used to detect protein expression.

### LC and MS analysis

For these studies, biological replicates for control diet (CD; *n*=4) and HFD (*n*=5) were examined. Protein isolation and enzymatic digestion – protein from LV tissue was homogenized, clarified, and quantitated, as described above in immunoblotting. Protein lysate was subsequently digested and desalted according to Lundby et al. [17] (*n*=5). Protein concentrations were then determined using BCA assay (Thermo Fisher Scientific, San Jose, CA). Proteins were then reduced and alkylated and subjected to methanol-chloroform precipitation prior to digestion with endoproteinase Lys-C (Wako, Richmond, VA). Followed by digestion with trypsin (Promega, Madison, WI) and desalting and concentration using C_18_ Sep-Pak Cartridges (Waters).

TMT isobaric labeling – thirty micrograms of peptides from each sample were mass tagged using Thermo Fisher’s TMT 10-plex isobaric label kit (catalog # 90061) following the included protocol and pooled for analysis; samples were normalized to sample 8, which had the lowest abundance on the peptide assay. Basic reversed-phase fractionation – pooled TMT-labeled peptides were fractionated by basic pH reversed-phase (BPRP) fractionation on an Ultimate 3000 HPLC (Thermo Scientific) using an integrated fraction collector. Elution was performed using a 10-min gradient of 0–20% solvent B followed by a 50-min gradient of solvent B from 20 to 45% (Solvent A 5.0% Acetonitrile, 10 mM ammonium bicarbonate pH 8.0, Solvent B 90.0% Acetonitrile, 10 mM ammonium bicarbonate pH 8.0) on a Zorbax 300Extend-C18 column (Agilent) at a flow rate of 0.4 ml/min. A total of 24 fractions were collected at 37-s intervals in a looping fashion for 60 min then combined to produce a total of 12 fractions. Peptide elution was monitored at a wavelength of 220 nm using a Dionex Ultimate 3000 variable wavelength detector (Thermo Scientific). Each fraction was then centrifuged to near dryness and desalted using C_18_ Sep-Pak Cartridges followed again by centrifugation to near dryness and reconstitution with 20 μl of 5% acetonitrile and 0.1% formic acid.

LC and MS – BPRP fractions were then separated using an UltiMate 3000 RSLCnano system (Thermo Scientific, San Jose, CA) on a self-packed UChrom C18 column (100 μm × 35 cm). Separation was performed using a 180-min gradient of solvent B from 2 to 27% (Solvent A 0.1% formic acid, Solvent B Acetonitrile, 0.1% formic acid) at 50°C using a digital Pico View nanospray source (New Objectives, Woburn, MA) that was modified with a custom-built column heater and an ABIRD background suppressor (ESI Source Solutions, Woburn, MA). Briefly, the self-packed column tapered tip was pulled with a laser micropipette puller P-2000 (Sutter Instrument Co, Novato, CA) to an approximate id of 10 μm. The column was then packed with 1–2 cm of 5 μm Sepax GP-C18 (120A) (Sepax Technologies, Newark, DE) followed by 40 cm of 1.8 μm Sepax GP-C18 (120 A) at 9000 psi using a nano LC column packing kit (nanoLCMS, Gold River, CA).

Mass spectral analysis was performed using an Orbitrap Fusion mass spectrometer (Thermo Scientific, San Jose, CA). TMT analysis was performed using an MS3 multi-notch approach [18]. The MS1 precursor selection range is from 400 to 1400 m/z at a resolution of 120 K and an automatic gain control (AGC) target of 2.0 × 10^5^ with a maximum injection time of 100 ms. Quadrupole isolation at 0.7 Th for MS^2^ analysis using CID fragmentation in the linear ion trap with a collision energy of 35%. The AGC was set to 4.0 × 10^3^ with a maximum injection time of 150 ms. The instrument was operated in a top speed data-dependent mode with a most intense precursor priority with dynamic exclusion set to an exclusion duration of 60 s with a 10 ppm tolerance. MS2 fragment ions were captured in the MS3 precursor population. These MS3 precursors were then isolated within a 2.5 Da window and subjected to high energy collision-induced dissociation (HCD) with a collision energy of 55%. The ions were then detected in the Orbitrap at a resolution of 60000 with an AGC of 5.0 × 10^4^ and a maximum injection time of 150 ms. The data were then analyzed using Sequest (Thermo Fisher Scientific, San Jose, CA, version v.27, rev. 11.) and Proteome Discoverer (Thermo Scientific, San Jose, CA, version 2.1).

### Peptide statistical analyses

Proteome Discoverer software was used to determine total protein and acetyl protein content within our samples. Search parameters were set for FASTA: Uniprot-*Mus musculus* (downloaded 7-17-2015) that contained 24088 sequences. The Proteome Discoverer peptide-level abundance data file was first transformed into a flat text file so that abundance data from each protein and its associated peptides were easily accessible for manipulation and analysis in the **R** programming language. More specifically, data from Proteome Discoverer was imported into Microsoft Access and SQL scripts were written to transform the data into a simple flat file so that each protein became a header for the subgroup of its associated peptides, and this row was clearly identified by its protein accession. Each peptide was represented by its (not necessarily unique) sequence, and two columns were included in the custom text file to clearly indicate whether the peptide was acetylated, and whether the Proteome Discoverer software used the peptide to account for the protein’s total abundance. For example, redundant peptides were not used to account for the protein’s total abundance. Additional information pertaining to methodology are highlighted in results.

### IPA

Proteins identified as being acetylated were analyzed using IPA software (Qiagen, Redwood City, CA) following methodology previously described [19]. IPA is a well-cited standalone pathway analysis tool that is commonly used to examine total protein expression; we considered this in our evaluation of protein acetylation. IPA was used for our 65 total proteins that changed with obesity and also for our 189 acetylated proteins. For both the total proteome and acetylated proteins, each protein symbol was mapped to its corresponding gene and set within the context of its associated partners based on the Ingenuity Pathways Knowledge Base (IPKD). Networks of these genes were algorithmically generated based on their connectivity and assigned a score. The score takes into account the number of focus genes in the network and the size of the network to approximate how relevant this network is to the original list of focus genes. Canonical pathways analysis identified the pathways, from the IPA library of canonical pathways, which were most significant to the input dataset. For acetylated peptides, post-hoc input was used to show proteins in which acetylation increased or decreased in response to DIO. Protein–protein interactions were based on total protein analysis.

### Data availability

Data will be made freely available upon request. The MS proteomics data in the present paper have been deposited in the ProteomeXchange Consortium (http://proteomecentral.proteomexchange.org) via the PRIDE partner repository [20]: dataset identifier PXD008385.

## Results

### Obesity-mediated cardiac remodeling

**Figure 1:**
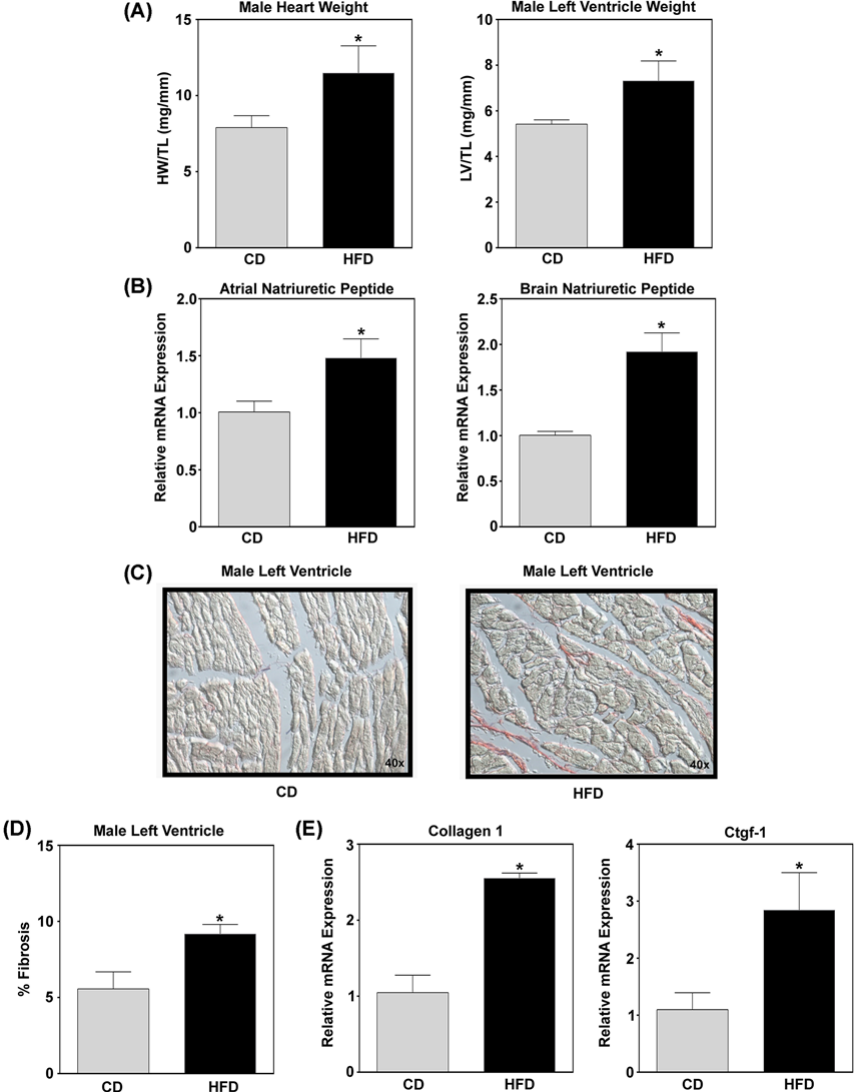
Obesity-mediated cardiac hypertrophy and fibrosis Hearts were extracted from male C57BL6/J mice on CD or HFD and (**A**) heart weight and LV weight measured and normalized to tibia length. (**B**) RNA was isolated from LV tissue and *ANP, BNP* mRNA expression was analyzed via qPCR from animals on CD and HFD. (**C**) PicroSirius Red staining of collagen in LV tissue of CD and HFD animals. (**D**) Quantitation of collagen, fibrosis, present in LV tissue of CD and HFD animals. At last, (**E**) mRNA expression of Col1 and Ctgf-1 was assessed by qPCR. Student’s *t* test with Welch’s correction using GraphPad Prism software was used to determine significance. *P<0.05.

The cardiovascular system adapts to stressors such as obesity, in part, through structural remodeling of the myocardium that is characterized by cardiac hypertrophy and fibrosis, as well as metabolic plasticity, which typically results in cardiac dysfunction and heart failure [2]. Thus, this study used a mouse model of obesity-mediated cardiac remodeling, as a pathophysiological relevant model of human dietary behavior. Sixteen weeks’ post-diet intervention, animals were killed, hearts were dissected and gross weights of heart weight and LV weight was assessed and normalized to tibia length. It should be noted that HFD animals were obese, hyperglycemic, and insulin resistant (data not shown) compared with their CD counterparts. Sixteen weeks of HFD led to a robust and significant increase in heart weight and LV weight (Figure 1A), demonstrating obesity-mediated increase in cardiac hypertrophy. Real-time qPCR of ANP and BNP, fetal genes that increase with cardiac enlargement and used as clinical indicators of heart failure, were additionally assessed in the LV. Similar to increased heart enlargement, animals in the HFD group had a significant induction of ANP and BNP gene expression (Figure 1B). Myocardial stress also typically results in cardiac fibrosis. Indeed, histological analysis of collagen stained LVs demonstrated a significant increase in cardiac fibrosis as assessed by PicroSirius Red staining (Figure 1C) and quantitated (Figure 1D) in animals under HFD conditions. These data were further supported, by gene expression analysis of collagen type 1 (Col1) and connective tissue growth factor 1 (Ctgf-1), genes that regulate extracellular matrix (ECM) production in the heart and are associated with fibrotic pathology [21]. Animals on the obesogenic diet (i.e. HFD) had a significant increase in *Col1* and *Ctgf-1* gene expression in the LV (Figure 1E). Together, these data demonstrate a robust and sufficient model for obesity-mediated cardiac remodeling in response to HFD.

### Protein lysine acetylation is regulated by DIO in the LV

**Figure 2:**
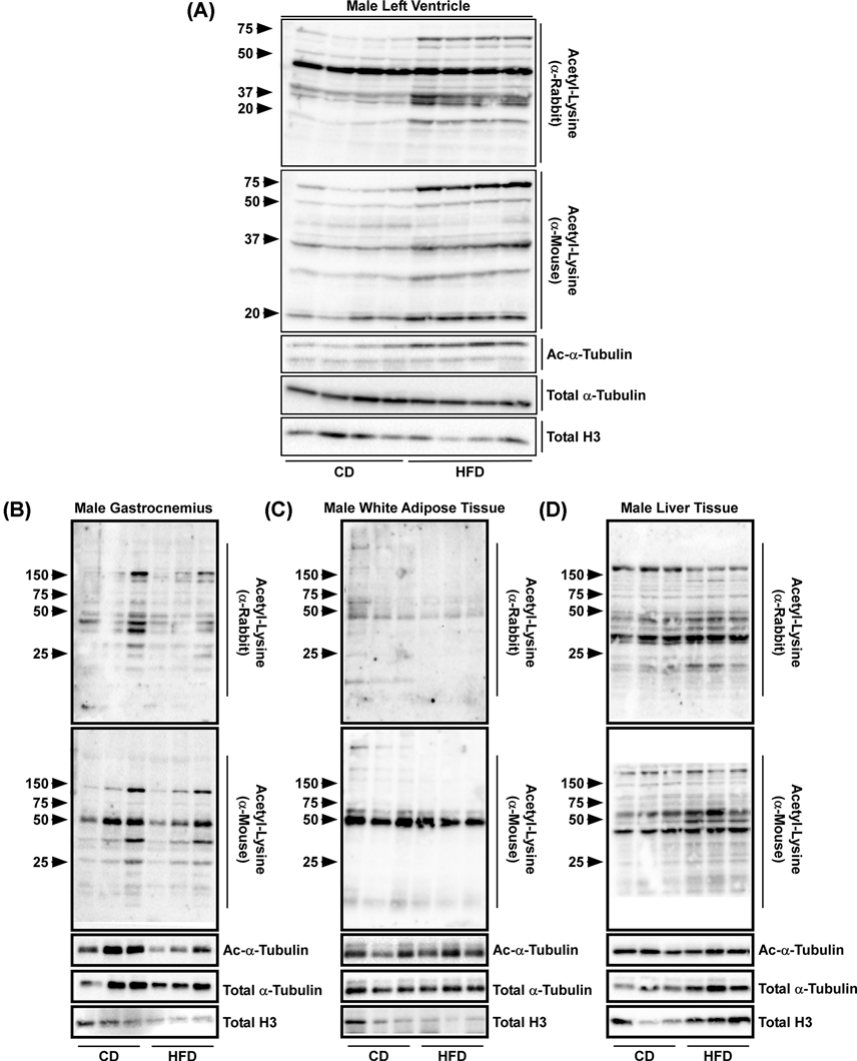
Obesity exacerbates cardiac tissue lysine acetylation Protein was isolated from (**A**) the LV, (**B**) gastrocnemius, (**C**) white epididymal adipose tissue, or (**D**) liver from mice fed a CD or HFD and immunoblotted for acetyl-lysine (α-rabbit), acetyl-lysine (α-mouse), acetyl-α-tubulin, total α-tubulin, and total H3.

Cardiac histone acetylation is a critical regulator of heart function, in which, changes in histone acetylation can contribute to cardiac hypertrophy and fibrosis, as well as loss of metabolic plasticity [5,22]. More recently however, investigators have begun to emphasize the impact of non-histone acetylation in biology, this postulate was strengthened with findings that approximately 4500 proteins can be acetylated [7]. Indeed, recent developments have shown that sarcomeric proteins can be acetylated and that this is important for cardiac function [9,10,23,24]. Given this, we first used standard SDS-PAGE and Western blot analysis to examine protein lysine acetylation. Animals on HFD had a significant increase in protein lysine acetylation in the LV compared with CD fed mice (Figure 2A). Two different acetyl lysine antibodies, mouse monoclonal and rabbit polyclonal, were used to verify this phenomenon. Of note, no changes in lysine protein acetylation were observed in the gastrocnemius, white adipose tissue (epididymal), or liver (Figure 2B–D, respectively), representing striated muscle and insulin-responsive tissues. These data suggest a cardiac tissue-specific phenomenon in animals exposed to 16 weeks of HFD.

### Obesity-mediated regulation of the cardiac proteome

**Figure 3:**
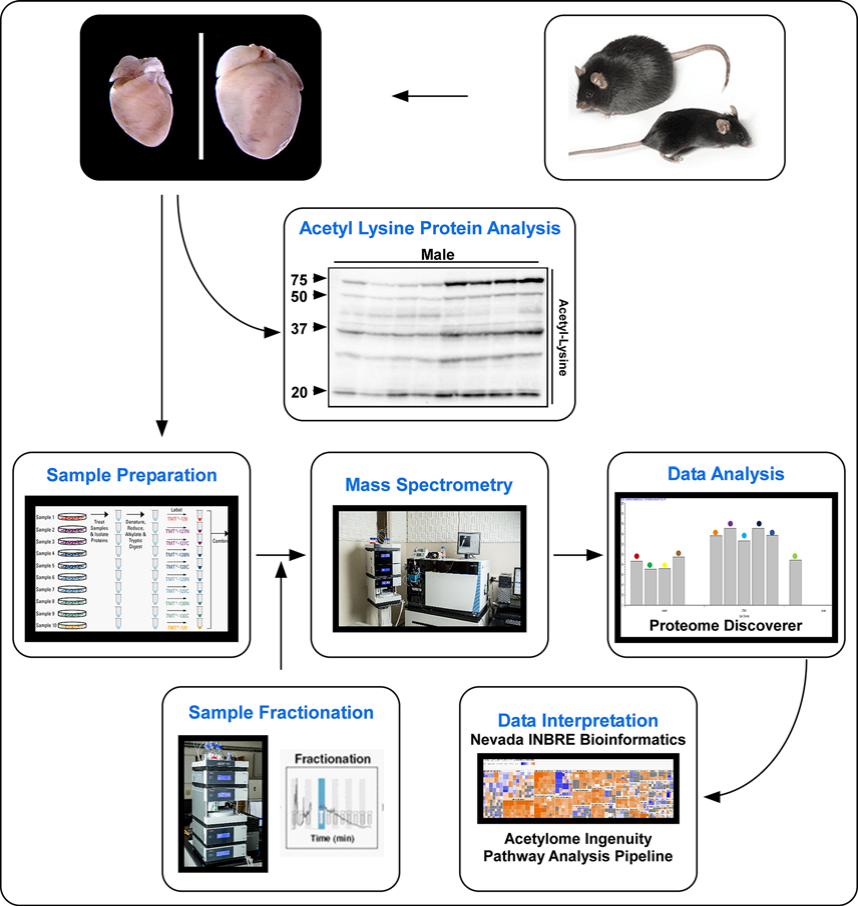
Experimental proteomic workflow to examine the cardiac acetylome Representation of the experimental workflow to examine the cardiac acetylome. Hearts from obese animal were dissected and protein lysates TMT tagged and assessed via tandem MS prior to data analysis, interpretation, and IPA.

Data presented above, demonstrate cardiac-specific regulation of lysine acetylation in response to DIO. As such, we next analyzed protein lysates from the LV of mice on a CD or HFD using LC MS analysis in order to identify and quantitate changes in total protein abundance and protein lysine acetylation. A workflow of the present study is summarized: (i) hearts were removed and LVs dissected from lean and obese mice in response to DIO, (ii) protein lysates were denatured, reduced, alkylated, and trypsin digested prior to TMT tagging, (iii) samples were then combined before sample fractionation and clean-up followed by (iv) MS/MS, (v) data were analyzed via Proteome Discoverer software, and (vi) bioinformatics analysis using IPA was performed (Figure 3). TMT-tagged MS was chosen over stable isotope labeling with amino acids in cell culture (SILAC) for total protein and acetyl protein quantitation. TMT-tagged MS allows for multiplex analysis (up to ten samples per run), compared with SILAC (up to four samples per run). In addition, TMT-tagged MS does not require *in vivo* labeling, compared with food enrichment necessary for SILAC. As such, TMT-tagged MS has reduced proteomic costs and improved statistical power.

TMT-tagged mass spectral analysis of four CD and five HFD mice LV protein samples identified a total of 4082 proteins. Abundance values obtained in all four CD and four out of five HFD cohorts met criteria for further study, which included 3928 of the 4082 identified proteins. Abundance values were normalized at the protein level, using the sum of the abundances of the pooled nine samples included on the multiplex. The average coefficient of variation (CV) across normalized protein abundances measured 25.3% for CD samples and 22.3% for HFD samples before quality control. A filtering step was used to reduce the overall CV in individual protein abundance values. Specifically, if exactly one outlier was 1.15 S.D. or more from the mean, it was removed. The maximum S.D. that is attainable in a sample with four replicates (CD cohort) is 1.5, and the maximum S.D. that is attainable in a sample with five replicates (HFD cohort) is 1.79; 15712 abundances were measured in the CD cohort and 20% of these were 1.15 S.D. or more from the mean protein abundance value. Similarly, 19640 abundances were measured in the HFD cohort and 14.4% of these were 1.15 S.D. or more from the mean protein abundance value and thus excluded. We found that these thresholds allow us to identify gross outlying individual data points within replicates and decrease the CV easily (only 17% of all data points were excluded) without excluding values of entire samples [25–29]. This simple quality control method identified grossly outlying replicates and decreased the average CV in the CD cohort to 14.8 and 15.3% in HFD cohort. Log_2_-transformed data followed a normal distribution.

Proteins were further filtered by only considering those with abundance levels for three out of four CD samples and three out of five HFD samples, and at least two peptides associated with the protein in each sample. This filtering protocol left 2515 proteins (Supplementary Table S1). Normalized data were first log_2_-transformed to follow a normal distribution. Simple Student’s *t* tests were performed to assess differences in means of abundance levels between CD and HFD cohorts. A correction for the false discovery rate on the 2515 hypothesis tests was made [30]. Sixty-five of the 2515 proteins showed statistically significant differences with adjusted *P*-values of *P*<0.1 (Supplementary Table S1). IPA determined these proteins to be involved in networks related to energy metabolism and production, cardiac necrosis and cardiovascular disease and endocrine function, with the top diseases related to metabolic disease and muscular disorders (Supplementary Table S2). In addition, IPA identified proteins from this screen that regulate cardiac enlargement, fibrosis, and heart failure.

**Figure 4:**
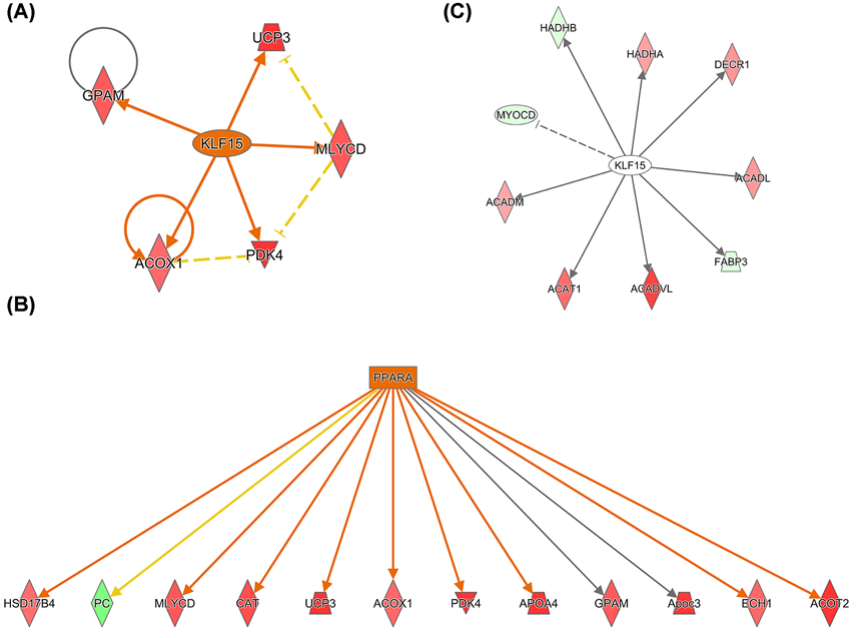
Obesity-mediated regulation of the cardiac proteome by KLF15 and PPARα transcriptional regulators (**A**,**B**) Pathway analysis (IPA) determined potential activation of the transcriptional regulators KLF15 and PPARα nuclear receptors by increased protein expression of downstream targets. Green highlighted proteins indicate a decrease in total protein expression and red indicate an increase in total protein expression. (**C**) IPA generated interaction map of KLF15 target proteins shown to be acetylated in this report. Green highlighted proteins indicate decreased acetylation and red indicate increased acetylation. Solid lines indicate direct protein–protein interactions, dashed lines indicate potential protein–protein interaction, and arrows imply directionality of protein–protein regulation.

Many of the proteins identified in the present study that showed increased expression with obesity are predominantly involved in energy utilization, either fatty acid β oxidation (e.g. malonyl-CoA decarboxylase (MLYCD)) or glucose oxidation (e.g. pyruvate dehydrogenase kinase isozyme 4 (PDK4)). For instance, IPA demonstrated both Kruppel-like factor 15 (KLF15) (Figure 4A) and peroxisome proliferator-activated receptor α (PPARα) (Figure 4B) to have a predicted activation based on downstream effectors showing increased protein expression. Proteins highlighted in red indicate increased protein expression and green indicate decreased protein expression impacted by obesity, where the predicted activated transcriptional regulators are highlighted in orange (Figure 4A,B). KLF15 is a transcriptional regulator known to negatively regulate cardiac remodeling in response to stress [31], such as pressure overload [32]. PPARα is a nuclear transcription factor that when activated by a lipophilic ligand, such as free fatty acids, forms a heterodimer with the retinoid X receptor (RXR) [33]. The activated heterodimer complex translocates into the nucleus and binds to target genes involved in fatty acid uptake, fatty acid storage, and fatty acid β oxidation [14,33].

### Obesity-mediated regulation of the cardiac acetylome

Similar to above, acetylated proteins were further filtered by considering those with abundance levels for three out of four CD samples and three out of five HFD samples, a minimum of two acetylated peptides associated with the protein in each sample were required for statistical analysis. This left 145 acetylated proteins (Supplementary Table S3). The same protein statistical analysis, performed above, was performed on the 145 acetylated proteins, 24 of the 145 acetylated proteins had *P*-values with *P*<0.05 (Supplementary Table S3, highlighted in orange), and five acetylated proteins had adjusted *P*-values with *P*<0.05 (Supplementary Table S3, highlighted in yellow). Principal Components (PCA) was performed on zero-centered, log_2_-transformed, normalized, and quality-controlled abundances on the 24 acetylated proteins using singular value decomposition, and showed a separation between high fat and control fat diets.

In addition to the above analyses, data of the four CD samples and five HFD samples were further extracted at the peptide level using Proteome Discoverer software. Using this software, 871 acetylated peptides were identified; 254 were unique peptides pertaining to a specific protein (Supplementary Table S4). The abundance of each acetylated peptide was normalized to the total abundance of its corresponding protein. This simple mathematical transformation provided a baseline of acetylated abundance for each peptide with respect to its protein. While enrichment is typical for examining protein modifications, our approach allowed for normalization of modified protein to its corresponding total protein with one simultaneous MS run. This limited our detection for low abundant acetylated proteins compared with standard enrichment. However, this approach of examining total protein lysate via TMT-tagged MS in combination with Proteome Discoverer software to determine peptide modification has the potential to limit technical biases associated with the combination of enrichment and MS analysis. This approach is additionally cost-effective as it limits multiple runs necessary for true acetyl/total protein normalization. Using this strategy, 189 unique acetylated proteins (Supplementary Table S5) were identified and the sum of the normalized abundances of the acetylated peptides associated with it was used for statistical testing across the CD and HFD cohorts. Specifically, simple Student’s *t* tests were applied to the log_2_-transformed sum of normalized abundance data of the acetylated peptides for each protein, and any test with resulting *P*-value *P*<0.1 was deemed statistically significant. This resulted in 14 acetylated proteins (Table 1) in which the acetylated peptide abundance showed a statistically significant difference between cohorts.

**Table 1:** Obesity-mediated cardiac protein acetylation (acetylated peptides normalized to total protein)

Protein	Accession	MW (kDa)	Modifications	Acetyl *P*-value
Hemoglobin subunit β-1 OS = *Mus musculus* GN = Hbb-b1 PE = 1 SV = 2	P02088	15.8	Acetyl (K18; S50; S51; S53; K60; K62; T140)	0.003634997
Hemoglobin subunit β-2 OS = *Mus musculus* GN = Hbb-b2 PE = 1 SV = 2	P02089	15.9	Acetyl (S53; K60; T140)	0.010349192
Polymerase I and transcript release factor OS = *Mus musculus* GN = Ptrf PE = 1 SV = 1	O54724	43.9	Acetyl (T5)	0.030179358
Peroxiredoxin-5: mitochondrial OS = *Mus musculus* GN = Prdx5 PE = 1 SV = 2	P99029	21.9	Acetyl (K70; K79)	0.03226413
Very long-chain specific acyl-CoA dehydrogenase: mitochondrial OS = *Mus musculus* GN = Acadvl PE = 1 SV = 3	P50544	70.8	Acetyl (K277; S306)	0.036561309
Dystonin OS = *Mus musculus* GN = Dst PE = 1 SV = 1	S4R1P5	870.0	Acetyl (K4661)	0.038409617
Aconitate hydratase: mitochondrial OS = *Mus musculus* GN = Aco2 PE = 1 SV = 1	Q99KI0	85.4	Acetyl (K144; K517; K523; K573)	0.046165279
Dihydrolipoyl dehydrogenase: mitochondrial OS = *Mus musculus* GN = Dld PE = 1 SV = 2	O08749	54.2	Acetyl (K159; T165; K166; K410)	0.058162495
Titin OS = *Mus musculus* GN = Ttn PE = 1 SV = 1	E9Q8K5	3713.7	Acetyl (T11523; K20887)	0.059301956
DnaJ homolog subfamily B member 6 OS = *Mus musculus* GN = Dnajb6 PE = 1 SV = 1	G3X8S5	29.5	Acetyl (S112; K134)	0.066036162
Myosin regulatory light chain 2: ventricular/cardiac muscle isoform OS = *Mus musculus* GN = Myl2 PE = 1 SV = 3	P51667	18.9	Acetyl (S19)	0.071717402
Actin: β skeletal muscle OS = *Mus musculus* GN = Acta1 PE = 1 SV = 1	P68134	42.0	Acetyl (K52; T150; T151; S273; T279; T280; S283; K317; T320; S325; T326; K328)	0.07295389
ADP-ribosylation factor-like protein 1 OS = *Mus musculus* GN = Arl1 PE = 2 SV = 1	P61211	20.4	Acetyl (S10; S11)	0.092968836
ATP synthase subunit O: mitochondrial OS = *Mus musculus* GN = Atp5o PE = 1 SV = 1	Q9DB20	23.3	Acetyl (K162; K172)	0.097114702

Abbreviation: Hbb-b1, hemoglobin subunit β-1.

From the 189 unique acetylated proteins identified (Supplementary Table S6), IPA demonstrated the top 15 canonical pathways significantly regulated by DIO (Figure 6A). Statistical significance was determined by -log(*P*-value) and color coding was used to represent proteins in which acetylation was down-regulated, up-regulated, or not changed with the percentage of these lysine acetylated proteins impacted by DIO within each canonical pathway shown (Figure 6A). Thus, while only 13.5% of the proteins in the mitochondrial dysfunction pathway were significantly impacted by obesity compared with the fatty acid β-oxidation pathway (~25%), the lower *P*-value suggested greater probability of finding proteins in the mitochondrial dysfunction pathway to be altered (Figure 6A). Indeed, of the 189 acetylated proteins, further stringent statistical parameters demonstrated that acetylation of 14 proteins were significantly regulated by DIO (Table 1), many of which are categorized in mitochondrial function (very long-chain specific Acyl-CoA dehydrogenase (ACADVL), aconitate hydratase 2 (ACO2), and dihydrolipoyl dehydrogenase (DLD)). From the 189 unique peptides identified (Supplementary Table S5) a *P*-value >0.05 and log_2_ (0.6) threshold suggested that nine proteins were deacetylated and five proteins acetylated, respectively, and no change in acetylation for 175 proteins in response to DIO (Figure 6B). Finally, using IPA, an overlapping canonical pathway map was generated (Figure 6C). Gray boxes represent the canonical pathways in Figure 6A, while black lines connecting the boxes reflect pathways that share at least ten acetylated proteins impacted by DIO (Figure 6C).

**Figure 5:**
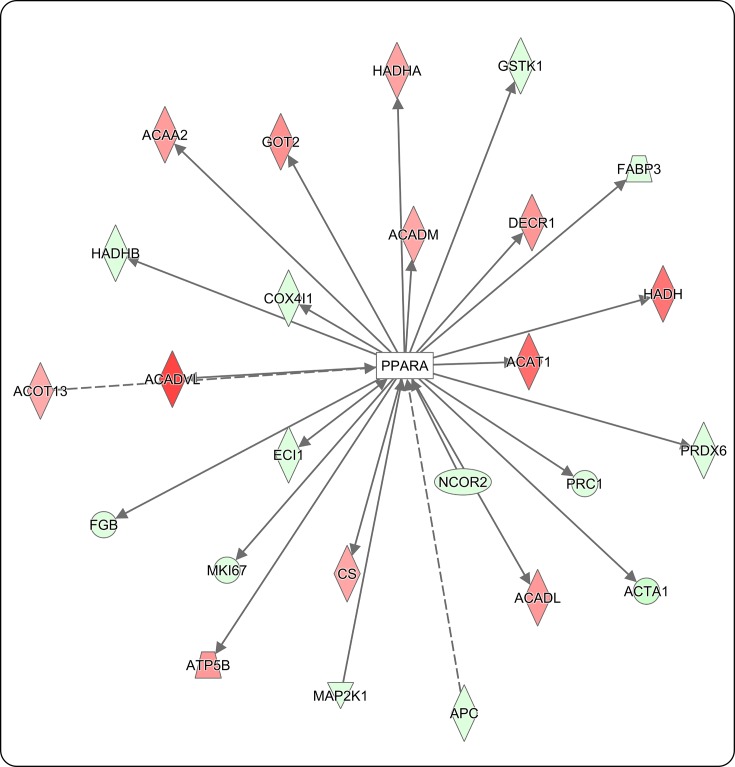
Obesity-mediated PPARα nuclear receptor regulation of the cardiac acetylome Pathway analysis (IPA) generated an interaction map of PPARα target proteins shown to be acetylated in this report. Green highlighted proteins indicate decreased acetylation and red indicate increased acetylation. Solid lines indicate direct protein–protein interactions, dashed lines indicate potential protein–protein interaction, and arrows imply directionality of protein–protein regulation.

With further examination, an overlay of acetylated proteins on the activated transcriptional regulators discussed above, KLF15 and PPARα, was performed. KLF15 is known to interact with nine of our identified acetylated proteins, including ACADVL (Figure 4C and Table 1). Proteins highlighted in red indicate increased acetylation and green indicate decreased acetylation impacted by obesity. Color intensity reflects the log_2_ fold change, with darker shades of red, for example demonstrating a larger increase in protein acetylation in response to DIO. In addition, this pathway highlights protein–protein interactions, in which solid lines reflect expression interactions in response to obesity; arrows indicate directionality of regulation, and no arrow indicates inhibition. KLF15 is known to induce expression of all proteins shown with an exception to inhibiting MYOCD (Figure 4C). PPARα is known to interact with 30 of our identified acetylated proteins, also including ACADVL, as well as α skeletal muscle actin (ACTA1) (Figure 5 and Table 1). Combined, these data suggest that protein acetylation has the potential to impact multiple pathways involved in cardiac cellular function in response to DIO.

### DIO impacts acetylation of proteins in the cardiovascular disease pathway

**Figure 6:**
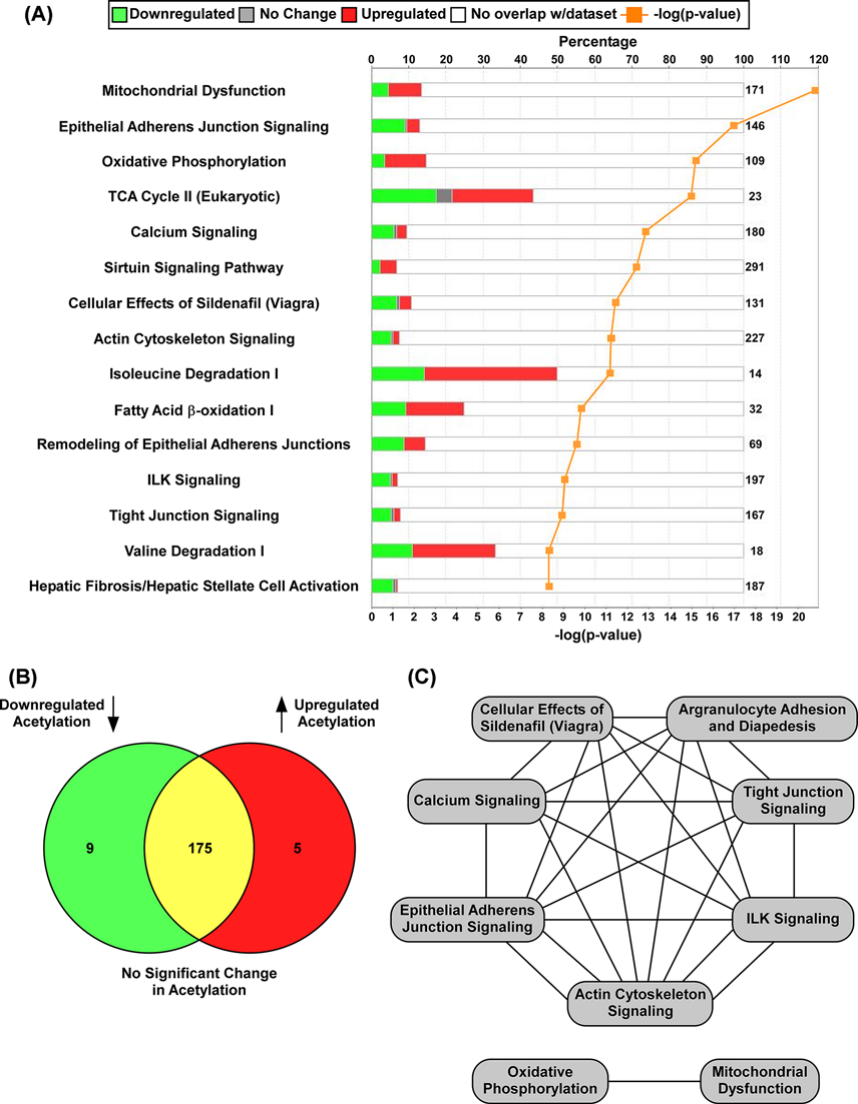
Obesity-mediated regulation of the cardiac acetylome and pathway analysis IPA generated (**A**) stacked canonical pathways significantly affected by obesity, (**B**) comparison of down- and up-regulated acetylated proteins, and (**C**) the overlapping pathways in which ten or more acetylated proteins are shared.

**Figure 7:**
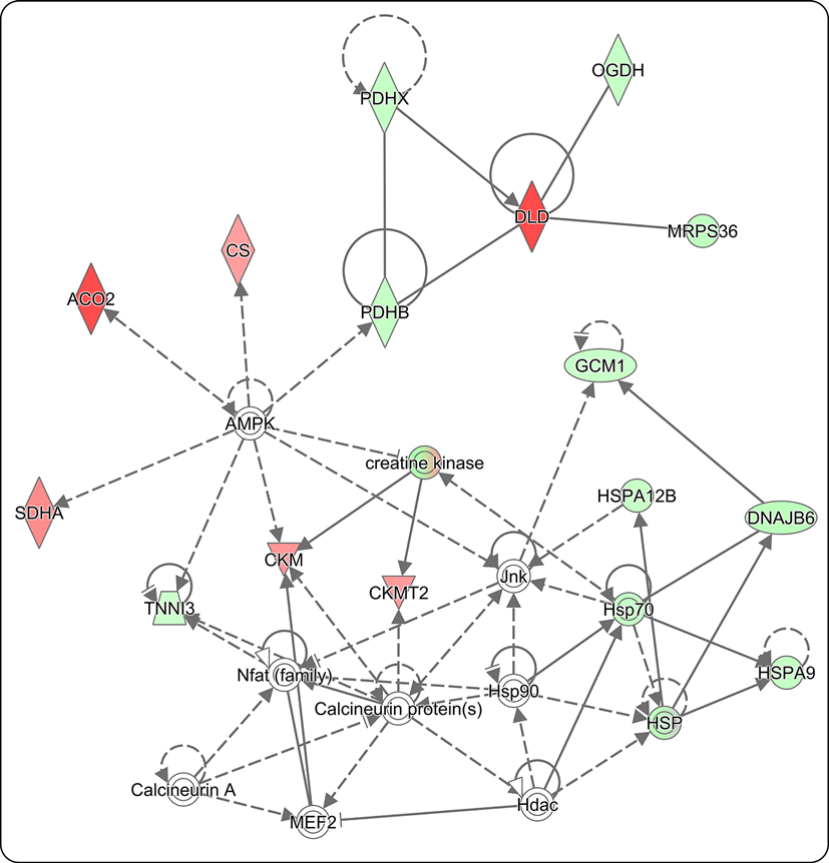
Protein acetylation impacts proteins involved in the cardiovascular disease pathway Down-regulation and up-regulation of protein acetylation within the Cardiovascular Disease Pathway impacted by obesity. Green highlighted proteins indicate decreased acetylation, red indicate increased acetylation, and white indicate no significant changes in acetylation. Solid lines indicate direct protein–protein interactions, dashed lines indicate potential protein–protein interaction, and arrows imply directionality of protein–protein regulation.

An IPA analysis report was performed, which outlines the top canonical pathways, diseases and functions by *P*-value on the proteins within our dataset to a related biological function. To summarize, the top five canonical pathways listed included Mitochondrial Dysfunction, Epithelial Adherens Junction Signaling, Oxidative Phosphorylation, TCA Cycle II (eukaryotic), and calcium signaling (Figure 6A and Supplementary Table S5). Furthermore, the top five diseases and biological functions included Cardiovascular Disease, Organismal Injury and Abnormalities, Skeletal and Muscular Disorders, Developmental Disorder, and Hereditary Disorder (Supplementary Table S6). The top disease listed, Cardiovascular Disease, had a calculated *P*-value range of (1.43E^−16^, 4.58E^−04^) and included 47 of the 189 acetylated proteins previously identified. The Cardiovascular Disease Pathway consists of a specific set of functions; in which each has an individual calculated *P*-value contributing to the *P*-value range of the Cardiovascular Disease Pathway. The acetylated proteins associated with a related function within the Cardiovascular Disease Pathway are shown (Figure 7). Proteins highlighted in red indicate increased acetylation and green indicate decreased acetylation impacted by obesity; proteins highlighted in white indicate no significant change. Color intensity reflects the log_2_ fold change, with darker shades of red indicating increased protein acetylation and bright green reflective of a greater decrease in protein acetylation in response to DIO (Figure 7). In addition, this pathway highlights protein–protein interactions, in which solid lines reflect known interactions whereas dashed lines suggest potential interactions in response to obesity; arrows indicate directionality of protein–protein regulation. Based on this knowledge, we can observe that several proteins in the Cardiovascular Disease Pathway are significantly acetylated in response to DIO that includes ACO2 and DLD (Figure 7 and Table 1). Additionally, we report that these proteins can interact with regulators of nutrient sensing (e.g. 5′-AMP activated protein kinase (AMPK)). Thus, these data imply that non-histone protein acetylation is important for obesity-mediated cardiac pathology.

## Discussion

Unlike standard enrichment methods that allow for detection of low abundant peptide modifications, this report used TMT-tagged MS of total protein lysate to demonstrate a significant impact of obesity on total protein and non-histone protein acetylation in the LVs of mice. This method limited acetyl peptide yield typically obtained with enrichment, yet afforded us the opportunity to normalize protein acetylation to its corresponding total protein in the same run; this allowed proper examination of acetyl protein changes in response to DIO. We present strong statistical evidence that obesity-mediated protein acetylation impacted proteins involved in energy metabolism and sarcomere function. These findings are consistent with recent reports noting significant changes in protein acetylation, where acetylation sites were predominantly observed on mitochondrial, sarcomeric, or cytoskeletal proteins [15,34,35]. Combined, these findings highlight the potential impact for the cardiac acetylome as a critical regulator of cardiac pathophysiology, with our data emphasizing cardiometabolic re-programming of non-histone acetyl marks that may function to disrupt energy utilization and sarcomere activity.

Obesity contributes to the development of metabolic syndrome, in which increased energy intake exceeds energy expenditure. This results in an excess of mitochondrial oxidation products that propagates mitochondrial dysfunction and insulin resistance, contributing to an increased risk of cardiovascular disease [36]. Indeed, mice in this study had increased enlargement and fibrosis of the heart (Figure 1). In addition, these animals were hyperglycemic and insulin resistant (data not shown). In response to metabolic dysfunction, we report that KLF15 and PPARα were predicted to have increased transcriptional activation, based on increased expression of downstream target genes (Figure 4A,B). KLF15 and PPARα act as nodal regulators of energy metabolism, in which deletion of PPARα/KLF15 shifts fuel utilization from fatty acid oxidation toward glucose oxidation [11]. The observation that KLF15 phenocopies PPARα in the regulation of cardiac metabolism has been supported by Prosdocimo et al. [37], where KLF15 was shown to bind to PPARα in order to regulate a subset of genes necessary for cardiac lipid metabolism. Indeed, we report transcriptional overlap between KLF15 and PPARα in the regulation of proteins involved in glucose oxidation (PDK4) and lipid metabolism (MLYCD) in the heart (Figure 4A,B, respectively). Increased PDK4 would be expected to attenuate glucose oxidation via inhibition of pyruvate dehydrogenase, while MLYCD would promote fatty acid oxidation via activation of carnitine palmitoyltransferase 1 (CPT1) [11,38,39]. Thus, these data would suggest disruption in metabolic homeostasis with increased nutrient demand; shifting toward fatty acid oxidation in place of glucose metabolism. This shift in metabolic homeostasis is common in states of metabolic dysfunction, where for instance in states of diabetes inhibition of insulin signaling shifts tissues toward fatty acid oxidation. Indeed, cardiac overexpression of PPARα mimics diabetic cardiomyopathy [40,41]. Combined, these data would suggest that DIO activates KLF15/PPARα in a manner that promotes increased expression of proteins leading to metabolic dysfunction during pathological cardiac remodeling.

While downstream targets of KLF15 and PPARα were impacted at the level of total protein expression (Figure 4A,B), proteins downstream of these nodal regulators also underwent PTMs that included acetylation/deacetylation (Figures 4C and 5). One such protein, ACADVL had increased protein acetylation in animals exposed to HFD. KLF15 and PPARα were previously reported to regulate ACADVL expression in cardiac tissue [11,33]. ACADVL catalyzes the first step in fatty acid β oxidation. Loss of ACADVL contributes to cardiomyopathy [42]. Interestingly, protein expression of ACADVL did not change in our DIO model, suggesting that acetylation may inhibit ACADVL activity and thus contribute to obesity-mediated metabolic dysfunction that contributes to cardiac remodeling and disease. Of note, ACADVL hyperacetylation was also reported in a transverse aortic constriction (TAC)-induced animal model of heart failure [34], further highlighting the potential role for ACADVL hyperacetylation in metabolic regulation. Studies have shown that sirtuin 3 (Sirt3), an NAD^+^-dependent HDAC, is important in cardiac disease, where loss of Sirt3 amplified cardiac remodeling [43]. This is of interest, as Sirt3 targets ACADVL for deacetylation [44]. Sirt3 expression is decreased with obesity [45]. These data would suggest that obesity-mediated down-regulation of Sirt3 potentially mediates ACADVL acetylation in the heart; whether KLF15/PPARα regulated Sirt3 in our model remains unknown. However, others have reported that Sirt3 co-localized with KLF15 in the nucleus of kidney cells [46]. Thus, KLF15/PPARα may interact with Sirt3 to regulate Sirt3 activity or expression in response to obesity and thus regulate ACADVL acetylation. These studies are currently underway.

Consistent with findings discussed above, we report that the Mitochondrial Dysfunction and TCA Cycle II (eukaryotic) pathways were two of the top three listed canonical pathways impacted by obesity (Figure 6A and Supplementary Table S6), where protein acetylation was altered in 13.5% of proteins involved in the mitochondrial dysfunction pathway and 43.5% of proteins involved in TCA Cycle II pathway. ACO2, for instance plays an important role in the TCA cycle, where it interconverts citrate into its isomer isocitrate. We report a significant increase in ACO2 acetylation in response to obesity. Consistent with our data, a recent report demonstrated a significant increase in mitochondrial ACO2 acetylation in the heart of animals exposed to an HFD [47]. Using molecular modeling, these investigators further suggested that acetylation of K144 on ACO2 could perturb its tertiary structure and thus alter its enzymatic function [47]. However, as of yet, a direct physiological consequence for ACO2 acetylation in the heart has not been reported. Dehydrogenases also play an important role in mitochondrial function, where for instance, mutations of the *DLD* gene are associated with branched-chain ketoaciduria and Leigh disease [48]. While it was previously reported that DLD is acetylated in HeLa cells and mouse liver [48], we report for the first time that DLD acetylation increased in the hearts of obese mice. Similar to ACO2, the functional consequence of obesity-mediated DLD acetylation remains unknown. Combined, however, these data suggest that ACO2 and DLD acetylation regulates mitochondrial dysfunction in cardiac tissue; thus, obesity-induced acetylation of ACO2 and DLD potentially promotes cardiac pathology. The role of non-histone acetylation of proteins involved in energy metabolism require further investigation to determine how these modifications link obesity to cardiovascular disease.

Changes for mitochondrial protein hyperacetylation, as discussed above, were observed in our rodent model of DIO. The DIO model leads to severe obesity, glucose intolerance, hyperglycemia, and moderate insulin resistance. Moreover, this model can contribute to increased circulating triglycerides and free fatty acids [49]. As mentioned, elevated circulating free fatty acids contribute to increased cardiac β oxidation, providing an abundance of acetyl-CoA molecules for protein lysine acetylation [13,14]. This would suggest that increased mitochondrial acetylation is unique to the obesity/diabetes phenotype. However, a recent report observed mitochondrial protein hyperacetylation in rodent models of heart failure and in end-stage failing human hearts [34], in which obesity and/or diabetes may be absent. Combined, this would suggest that mitochondrial protein hyperacetylation is an important regulator of metabolic dysfunction that has been linked to cardiac disease and is not dependent on metabolic phenotype. In that report [34], investigators performed mitochondrial fractionation studies from cardiac tissue to elucidate mitochondrial-specific protein targets. While our studies failed to use fractionated lysate, proteins identified in our analysis were shown in that report to locate to the mitochondria. Indeed, many of our acetylated proteins have been reported to localize to the mitochondria (e.g. ACO2) or the sarcomere (e.g. Titin), with additional proteins identified as circulating factors within the blood (i.e. hemoglobin). As the mitochondrial hyperacetylation report [34] did not examine sarcomeric proteins or circulating factors, it is possible that acetylation changes observed with these proteins are specific to the obesity/diabetes phenotype and therefore would imply that future studies examining protein acetylation in other compartments in response to cardiac stress are needed.

At last, comparisons between this report and previous investigations noted acetylation/deacetylation for many of the same proteins including ACO2, DLD, ACADVL, ACTA1, and LIM domain binding protein 3 (LDB3), with similarities and differences noted for sites of acetylation [7,34]. For example, we report similar findings to Lundby et al. [7], in which DLD was acetylated on lysine residues 159 (K159) and 410 (K410) in the heart. Of interest, Lundby et al. [7] reported acetylation of hemoglobin subunit β-1 (Hbb-b1) in rat heart tissue at lysine residues 18 (K18) and 62 (K62). Similarly, we observed acetylation of Hbb-b1 at K18 and K62 in control mice, yet Hbb-b1 acetylation decreased in our DIO mice. In addition to Hbb-b1, we further observed deacetylation of hemoglobin subunit β-2 in the hearts of DIO mice. Findings that circulating proteins can be acetylated or deacetylated suggest that examination of acetyl-hemoglobin proteins or other acetylated circulating factors have the potential to serve as biomarkers for early cardiovascular disease detection. Indeed, many protein biomarkers serve for the evaluation and management of myocardial insults and cardiac remodeling; these include BNP, N-terminal proBNP, C-reactive protein, and Troponin T amongst others [50,51]. In addition, hemoglobin content combined with serum folate levels have been suggested as potential biomarkers for Alzheimer’s disease [52]. Combined, this would support further investigation into the role for acetylated or deacetylated circulating factors as biomarkers for cardiovascular disease; blood analysis from patients serves as a relatively simple and non-invasive procedure in the clinic.

In summary, our data highlight a potential role for non-histone protein acetylation in cardiac pathology. Further mechanistic investigation regarding the impact for protein acetylation on energy metabolism and sarcomeric function in response to physiological and pathological stress will yield novel insights in cardiac biology. Many of these mechanistic investigations are currently underway. At last, perturbations in acetylation are reversible, thus mechanistic insight regarding the cardiac acetylome may uncover novel therapeutic targets for the treatment of cardiometabolic disease.

## Supporting information

**Supplemental Table 1.** The cardiac proteome.

**Supplemental Table 2.** Total Protein Top Diseases, Disorders, and Toxicity

**Supplemental Table 3.** The cardiac acetylome.

**Supplemental Table 4.** Unique Acetylated Peptides

**Supplemental Table 5.** Cardiac Acetylated Proteins

**Supplemental Table 6.** Acetylated Protein Top Diseases, Disorders, and Toxicity

## Abbreviations

ACADVL: very long-chain specific Acyl-CoA dehydrogenase
ACO2: aconitate hydratase 2
ACTA1: α skeletal muscle actin
AGC: automatic gain control
ANP: atrial natriuretic peptide
BNP: brain natriuretic peptide
BPRP: basic pH reversed-phase
CD: control diet
Col1: collagen type 1
Ctgf-1: connective tissue growth factor 1
CV: coefficient of variation
DIO: diet-induced obesity
DLD: dihydrolipoyl dehydrogenase
Hbb-b1: hemoglobin subunit β-1
HDAC: histone deacetylase
HFD: high-fat diet
IPA: Ingenuity Pathway Analysis
KLF15: Kruppel-like factor 15
LV: left ventricle
MLYCD: malonyl-CoA decarboxylase
PDK4: pyruvate dehydrogenase kinase isozyme 4
PPARα: peroxisome proliferator-activated receptor α
PTM: post-translational modification
qPCR: quantitative PCR
qRT-PCR: quantitative real-time polymerase chain reaction
SILAC: stable isotope labeling with amino acids in cell culture
Sirt3: sirtuin 3
TMT: tandem mass tag

## References

[1] Smith K.B. and Smith M.S. (2016) Obesity statistics. Prim. Care 43, 121–135, 10.1016/j.pop.2015.10.001 26896205

[2] Aurigemma G.P., de Simone G. and Fitzgibbons T.P. (2013) Cardiac remodeling in obesity. Circ. Cardiovasc. Imaging 6, 142–152 10.1161/CIRCIMAGING.111.964627 23322729

[3] Barry S.P., Davidson S.M. and Townsend P.A. (2008) Molecular regulation of cardiac hypertrophy. Int. J. Biochem. Cell Biol. 40, 2023–2039 10.1016/j.biocel.2008.02.020 18407781

[4] Miyata S., Minobe W., Bristow M.R. and Leinwand L.A. (2000) Myosin heavy chain isoform expression in the failing and nonfailing human heart. Circ. Res. 86, 386–390 10.1161/01.RES.86.4.386 10700442

[5] Stratton M.S. and McKinsey T.A. (2016) Epigenetic regulation of cardiac fibrosis. J. Mol. Cell Cardiol. 92, 206–213 10.1016/j.yjmcc.2016.02.011 26876451 PMC4987078

[6] Ferguson B.S. and McKinsey T.A. (2015) Non-sirtuin histone deacetylases in the control of cardiac aging. J. Mol. Cell Cardiol. 83, 14–20 10.1016/j.yjmcc.2015.03.010 25791169 PMC4459895

[7] Lundby A., Lage K., Weinert B.T., Bekker-Jensen D.B., Secher A., Skovgaard T. (2012) Proteomic analysis of lysine acetylation sites in rat tissues reveals organ specificity and subcellular patterns. Cell Rep. 2, 419–431 10.1016/j.celrep.2012.07.006 22902405 PMC4103158

[8] Samant S.A., Pillai V.B., Sundaresan N.R., Shroff S.G. and Gupta M.P. (2015) Histone deacetylase 3 (HDAC3)-dependent reversible lysine acetylation of cardiac myosin heavy chain isoforms modulates their enzymatic and motor activity. J. Biol. Chem. 290, 15559–15569 10.1074/jbc.M115.653048 25911107 PMC4505469

[9] Demos-Davies K.M., Ferguson B.S., Cavasin M.A., Mahaffey J.H., Williams S.M., Spiltoir J.I. (2014) HDAC6 contributes to pathological responses of heart and skeletal muscle to chronic angiotensin-II signaling. Am. J. Physiol. Heart Circ. Physiol. 307, H252–H258 10.1152/ajpheart.00149.2014 24858848 PMC4101640

[10] Zhang D., Wu C.T., Qi X., Meijering R.A., Hoogstra-Berends F., Tadevosyan A. (2014) Activation of histone deacetylase-6 induces contractile dysfunction through derailment of alpha-tubulin proteostasis in experimental and human atrial fibrillation. Circulation 129, 346–358 10.1161/CIRCULATIONAHA.113.005300 24146251

[11] Prosdocimo D.A., Anand P., Liao X., Zhu H., Shelkay S., Artero-Calderon P. (2014) Kruppel-like factor 15 is a critical regulator of cardiac lipid metabolism. J. Biol. Chem. 289, 5914–5924 10.1074/jbc.M113.531384 24407292 PMC3937660

[12] Mazumder P.K., O’Neill B.T., Roberts M.W., Buchanan J., Yun U.J., Cooksey R.C. (2004) Impaired cardiac efficiency and increased fatty acid oxidation in insulin-resistant ob/ob mouse hearts. Diabetes 53, 2366–2374 10.2337/diabetes.53.9.2366 15331547

[13] Pougovkina O., te Brinke H., Ofman R., van Cruchten A.G., Kulik W., Wanders R.J. (2014) Mitochondrial protein acetylation is driven by acetyl-CoA from fatty acid oxidation. Hum. Mol. Genet. 23, 3513–3522 10.1093/hmg/ddu059 24516071

[14] Lopaschuk G.D., Ussher J.R., Folmes C.D., Jaswal J.S. and Stanley W.C. (2010) Myocardial fatty acid metabolism in health and disease. Physiol. Rev. 90, 207–258 10.1152/physrev.00015.2009 20086077

[15] Foster D.B., Liu T., Rucker J., O’Meally R.N., Devine L.R., Cole R.N. (2013) The cardiac acetyl-lysine proteome. PLoS ONE 8, e67513 10.1371/journal.pone.0067513 23844019 PMC3699649

[16] Williams S.M., Golden-Mason L., Ferguson B.S., Schuetze K.B., Cavasin M.A., Demos-Davies K. (2014) Class I HDACs regulate angiotensin II-dependent cardiac fibrosis via fibroblasts and circulating fibrocytes. J. Mol. Cell Cardiol. 67, 112–125 10.1016/j.yjmcc.2013.12.013 24374140 PMC4120952

[17] Lundby A., Secher A., Lage K., Nordsborg N.B., Dmytriyev A., Lundby C. (2012) Quantitative maps of protein phosphorylation sites across 14 different rat organs and tissues. Nat. Commun. 3, 876 10.1038/ncomms1871 22673903 PMC3621391

[18] McAlister G.C., Nusinow D.P., Jedrychowski M.P., Wuhr M., Huttlin E.L., Erickson B.K. (2014) MultiNotch MS3 enables accurate, sensitive, and multiplexed detection of differential expression across cancer cell line proteomes. Anal. Chem. 86, 7150–7158 10.1021/ac502040v 24927332 PMC4215866

[19] Kramer A., Green J., Pollard J. Jr and Tugendreich S. (2014) Causal analysis approaches in Ingenuity Pathway Analysis. Bioinformatics 30, 523–530 10.1093/bioinformatics/btt703 24336805 PMC3928520

[20] Vizcaino J.A., Csordas A., Del-Toro N., Dianes J.A., Griss J., Lavidas I. (2016) 2016 update of the PRIDE database and its related tools. Nucleic Acids Res. 44, 11033 27683222 10.1093/nar/gkw880 PMC5159556

[21] Brigstock D.R. (2010) Connective tissue growth factor (CCN2, CTGF) and organ fibrosis: lessons from transgenic animals. J. Cell Commun. Signal 4, 1–4 10.1007/s12079-009-0071-5 19798591 PMC2821473

[22] McKinsey T.A. (2011) Isoform-selective HDAC inhibitors: closing in on translational medicine for the heart. J. Mol. Cell Cardiol. 51, 491–496 10.1016/j.yjmcc.2010.11.009 21108947

[23] Samant S.A., Courson D.S., Sundaresan N.R., Pillai V.B., Tan M., Zhao Y. (2011) HDAC3-dependent reversible lysine acetylation of cardiac myosin heavy chain isoforms modulates their enzymatic and motor activity. J. Biol. Chem. 286, 5567–5577 10.1074/jbc.M110.163865 21177250 PMC3037670

[24] Gupta M.P., Samant S.A., Smith S.H. and Shroff S.G. (2008) HDAC4 and PCAF bind to cardiac sarcomeres and play a role in regulating myofilament contractile activity. J. Biol. Chem. 283, 10135–10146 10.1074/jbc.M710277200 18250163 PMC2442284

[25] Aw T., Schlauch K., Keeling C.I., Young S., Bearfield J.C., Blomquist G.J. (2010) Functional genomics of mountain pine beetle (Dendroctonus ponderosae) midguts and fat bodies. BMC Genomics 11, 215 10.1186/1471-2164-11-215 20353591 PMC2858752

[26] Miller G., Schlauch K., Tam R., Cortes D., Torres M.A., Shulaev V. (2009) The plant NADPH oxidase RBOHD mediates rapid systemic signaling in response to diverse stimuli. Sci. Signal. 2, ra45, 10.1126/scisignal.2000448 19690331

[27] Altick A.L., Feng C.Y., Schlauch K., Johnson L.A. and von Bartheld C.S. (2012) Differences in gene expression between strabismic and normal human extraocular muscles. Invest. Ophthalmol. Vis. Sci. 53, 5168–5177 10.1167/iovs.12-9785 22786898 PMC3416046

[28] Fennell A.Y., Schlauch K.A., Gouthu S., Deluc L.G., Khadka V., Sreekantan L. (2015) Short day transcriptomic programming during induction of dormancy in grapevine. Front. Plant Sci. 6, 834 10.3389/fpls.2015.00834 26582400 PMC4632279

[29] Kuhn A.R., Schlauch K., Lao R., Halayko A.J., Gerthoffer W.T. and Singer C.A. (2010) MicroRNA expression in human airway smooth muscle cells: role of miR-25 in regulation of airway smooth muscle phenotype. Am. J. Respir. Cell Mol. Biol. 42, 506–513 10.1165/rcmb.2009-0123OC 19541842 PMC2848741

[30] Benjamini Y. and Hochberg Y. (1995) Controlling the false discovery rate - a practical and powerful approach to multiple testing. J. Roy. Stat. Soc. B. Met. 57, 289–300

[31] Fisch S., Gray S., Heymans S., Haldar S.M., Wang B., Pfister O. (2007) Kruppel-like factor 15 is a regulator of cardiomyocyte hypertrophy. Proc. Natl. Acad. Sci. U.S.A. 104, 7074–7079 10.1073/pnas.0701981104 17438289 PMC1855421

[32] Yu Y., Ma J., Xiao Y., Yang Q., Kang H., Zhen J. (2015) KLF15 is an essential negative regulatory factor for the cardiac remodeling response to pressure overload. Cardiology 130, 143–152 10.1159/000369382 25633973

[33] Francis G.A., Fayard E., Picard F. and Auwerx J. (2003) Nuclear receptors and the control of metabolism. Annu. Rev. Physiol. 65, 261–311 10.1146/annurev.physiol.65.092101.142528 12518001

[34] Horton J.L., Martin O.J., Lai L., Riley N.M., Richards A.L., Vega R.B. (2016) Mitochondrial protein hyperacetylation in the failing heart. JCI Insight 2, 26998524 10.1172/jci.insight.84897 PMC4795836

[35] Davies M.N., Kjalarsdottir L., Thompson J.W., Dubois L.G., Stevens R.D., Ilkayeva O.R. (2016) The acetyl group buffering action of carnitine acetyltransferase offsets macronutrient-induced lysine acetylation of mitochondrial proteins. Cell Rep. 14, 243–254 10.1016/j.celrep.2015.12.030 26748706 PMC4754083

[36] Reaven G.M. (2011) Insulin resistance: the link between obesity and cardiovascular disease. Med. Clin. North Am. 95, 875–892 10.1016/j.mcna.2011.06.002 21855697

[37] Prosdocimo D.A., John J.E., Zhang L., Efraim E.S., Zhang R., Liao X. (2015) KLF15 and PPARalpha cooperate to regulate cardiomyocyte lipid gene expression and oxidation. PPAR Res. 2015, 201625 10.1155/2015/201625 25815008 PMC4357137

[38] Kwon H.S., Huang B., Ho Jeoung N., Wu P., Steussy C.N. and Harris R.A. (2006) Retinoic acids and trichostatin A (TSA), a histone deacetylase inhibitor, induce human pyruvate dehydrogenase kinase 4 (PDK4) gene expression. Biochim. Biophys. Acta 1759, 141–151 10.1016/j.bbaexp.2006.04.005 16757381

[39] Koves T.R., Ussher J.R., Noland R.C., Slentz D., Mosedale M., Ilkayeva O. (2008) Mitochondrial overload and incomplete fatty acid oxidation contribute to skeletal muscle insulin resistance. Cell Metab. 7, 45–56 10.1016/j.cmet.2007.10.013 18177724

[40] Finck B.N., Lehman J.J., Leone T.C., Welch M.J., Bennett M.J., Kovacs A. (2002) The cardiac phenotype induced by PPARalpha overexpression mimics that caused by diabetes mellitus. J. Clin. Invest. 109, 121–130 10.1172/JCI0214080 11781357 PMC150824

[41] Finck B.N., Han X., Courtois M., Aimond F., Nerbonne J.M., Kovacs A. (2003) A critical role for PPARalpha-mediated lipotoxicity in the pathogenesis of diabetic cardiomyopathy: modulation by dietary fat content. Proc. Natl. Acad. Sci. U.S.A. 100, 1226–1231 10.1073/pnas.0336724100 12552126 PMC298755

[42] Strauss A.W., Powell C.K., Hale D.E., Anderson M.M., Ahuja A., Brackett J.C. (1995) Molecular basis of human mitochondrial very-long-chain acyl-CoA dehydrogenase deficiency causing cardiomyopathy and sudden death in childhood. Proc. Natl. Acad. Sci. U.S.A. 92, 10496–10500 10.1073/pnas.92.23.10496 7479827 PMC40638

[43] Wei T., Huang G., Gao J., Huang C., Sun M., Wu J. (2017) Sirtuin 3 deficiency accelerates hypertensive cardiac remodeling by impairing angiogenesis. J. Am. Heart Assoc. 6, 10.1161/JAHA.117.006114 PMC5586452 28862956

[44] Bharathi S.S., Zhang Y., Mohsen A.W., Uppala R., Balasubramani M., Schreiber E. (2013) Sirtuin 3 (SIRT3) protein regulates long-chain acyl-CoA dehydrogenase by deacetylating conserved lysines near the active site. J. Biol. Chem. 288, 33837–33847 10.1074/jbc.M113.510354 24121500 PMC3837126

[45] Kendrick A.A., Choudhury M., Rahman S.M., McCurdy C.E., Friederich M., Van Hove J.L. (2011) Fatty liver is associated with reduced SIRT3 activity and mitochondrial protein hyperacetylation. Biochem. J. 433, 505–514 10.1042/BJ20100791 21044047 PMC3398511

[46] Li N., Zhang J., Yan X., Zhang C., Liu H., Shan X. (2017) SIRT3-KLF15 signaling ameliorates kidney injury induced by hypertension. Oncotarget 8, 39592–395604 28465484 10.18632/oncotarget.17165 PMC5503635

[47] Fernandes J., Weddle A., Kinter C.S., Humphries K.M., Mather T., Szweda L.I. (2015) Lysine acetylation activates mitochondrial aconitase in the heart. Biochemistry 54, 4008–4018 10.1021/acs.biochem.5b00375 26061789 PMC4513942

[48] Kim S.C., Sprung R., Chen Y., Xu Y., Ball H., Pei J. (2006) Substrate and functional diversity of lysine acetylation revealed by a proteomics survey. Mol. Cell 23, 607–618 10.1016/j.molcel.2006.06.026 16916647

[49] Kleinert M., Clemmensen C., Hofmann S.M., Moore M.C., Renner S., Woods S.C. (2018) Animal models of obesity and diabetes mellitus. Nat. Rev. Endocrinol. 14, 140–162 10.1038/nrendo.2017.161 29348476

[50] Gaggin H.K. and Januzzi J.L. Jr (2013) Biomarkers and diagnostics in heart failure. Biochim. Biophys. Acta. 1832, 2442–2450 10.1016/j.bbadis.2012.12.014 23313577

[51] Motiwala S.R., Szymonifka J., Belcher A., Weiner R.B., Baggish A.L., Gaggin H.K. (2014) Measurement of novel biomarkers to predict chronic heart failure outcomes and left ventricular remodeling. J. Cardiovasc. Transl. Res. 7, 250–261 10.1007/s12265-013-9522-8 24309956

[52] Yoshinaga T., Nishimata H., Kajiya Y. and Yokoyama S. (2017) Combined assessment of serum folate and hemoglobin as biomarkers of brain amyloid beta accumulation. PLoS ONE 12, e0175854 10.1371/journal.pone.0175854 28406978 PMC5391131

